# Diagnostic potential of urinary CX3CL1 for amnestic mild cognitive impairment and Alzheimer's disease

**DOI:** 10.1002/alz70856_102347

**Published:** 2025-12-25

**Authors:** Yali Xu

**Affiliations:** ^1^ Chongqing General Hospital, Chongqing University, Chongqing, Chongqing, China

## Abstract

**Background:**

Inflammation plays an integral role in Alzheimer's disease (AD), and chemokine CX3CL1 (C‐X3‐C motif ligand 1, aka Fractalkine), emerging as a key molecule linking inflammatory and neuroprotective mechanisms within the central nervous system, play a special role in the development of AD and ageing. Previous studies have focused on changes in CX3CL1 levels in biological samples such as blood or cerebrospinal fluid (CSF). However, the value of urine, as a relatively easy‐to‐obtain and non‐invasive biological sample, in reflecting dynamic changes in CX3CL1 in vivo remains to be comprehensively investigated. This study aims to evaluate the diagnostic potential of urinary CX3CL1 levels in distinguishing between AD patients, those experiencing amnestic mild cognitive impairment (aMCI), and cognitively unimpaired (CU) individuals.

**Method:**

A cohort comprising 516 cognitively unimpaired (CU) individuals across various age groups, 102 AD patients, and 65 subjects with aMCI was assembled, alongside 93 age‐ and sex‐matched CN controls. Enzyme‐linked immunosorbent assay (ELISA) was utilized and urinary creatine levels were normalized to quantify urinary CX3CL1 levels.

**Result:**

Urinary CX3CL1 concentrations exhibited an age‐dependent increase and demonstrated a positive correlation with age. Comparatively, AD patients exhibited significantly elevated urinary CX3CL1 levels when contrasted with both the CU controls and the aMCI cohort. Conversely, aMCI patients displayed urinary CX3CL1 levels that were notably reduced in comparison to both the AD and CU groups.

**Conclusion:**

Urinary CX3CL1 levels correlate with the aging process and may serve as a potential diagnostic biomarker for both amnestic mild cognitive impairment (aMCI) and Alzheimer's disease (AD).

